# Floating Liver

**Published:** 1899-10-07

**Authors:** 


### FLOATING LIVER.

Displacement of the liver, hepatoptosis, or, as it is sometimes called, floating liver, is a condition which is probably much more common than is generally supposed. The whole subject has recently been ably discussed by Dr. Max Einhorn,[1] who describes several cases. He says that the diagnosis of floating liver is based on the demonstration of the fact that the organ has left its normal limits in the thorax and has been displaced more or less forwards and downwards, without the existence of any disorder in the thorax to push it down. Difficulty of diagnosis has, however, been met with in case of hepatic tumours, and in some cases the displaced liver has been mistaken for renal tumour or hydronephrosis. The treatment is the same as that of floating kidney or of enteroptosis. Bandage to the lower half of the abdomen, general massage and hydrotherapeutic measures, diet, gymnastic exercises in the open air, and special exercises for the abdominal muscles are all useful, and such measures are recommended in preference to operative proceedings. It is also pointed out that the condition is by no means an uncommon one, and that when it gives rise to colic it may easily be confounded with cholelithiasis, while, unless the physical examination is carefully conducted so as to detect the real condition, the almost constant pain and the presence of a tumour below the ribs may lead to a wrong diagnosis of cancer of the stomach.

[1] New York Med. Rec., Sept. 16.

